# Sleep duration over 28 years, cognition, gray matter volume, and white matter microstructure: a prospective cohort study

**DOI:** 10.1093/sleep/zsz290

**Published:** 2020-01-06

**Authors:** Jennifer Zitser, Melis Anatürk, Enikő Zsoldos, Abda Mahmood, Nicola Filippini, Sana Suri, Yue Leng, Kristine Yaffe, Archana Singh-Manoux, Mika Kivimaki, Klaus Ebmeier, Claire Sexton

**Affiliations:** 1 Department of Neurology, Global Brain Health Institute, Memory and Aging Center, University of California, San Francisco, CA; 2 Department of Neurology, Movement Disorders Unit, Tel Aviv Sourazky Medical Center, Affiliated to the Sackler Faculty of Medicine, Tel-Aviv University, Israel; 3 Department of Psychiatry, University of Oxford, Oxford, UK; 4 Oxford Centre for Human Brain Activity, Wellcome Centre for Integrative Neuroimaging, Department of Psychiatry, University of Oxford, Oxford, UK; 5 FMRIB, Wellcome Centre for Integrative Neuroimaging, University of Oxford, Oxford, UK; 6 Department of Psychiatry, University of California, San Francisco, CA; 7 Department of Psychiatry, Neurology and Epidemiology, University of California, San Francisco, CA; 8 Université de Paris, Inserm U1153, Epidemiology of Ageing and Neurodegenerative Diseases, Paris, France; 9 Department of Epidemiology and Public Health, University College London, London, UK

**Keywords:** aging, cognition, gray matter, sleep, white matter

## Abstract

**Study Objectives:**

To examine the association between sleep duration trajectories over 28 years and measures of cognition, gray matter volume, and white matter microstructure. We hypothesize that consistently meeting sleep guidelines that recommend at least 7 hours of sleep per night will be associated with better cognition, greater gray matter volumes, higher fractional anisotropy, and lower radial diffusivity values.

**Methods:**

We studied 613 participants (age 42.3 ± 5.03 years at baseline) who self-reported sleep duration at five time points between 1985 and 2013, and who had cognitive testing and magnetic resonance imaging administered at a single timepoint between 2012 and 2016. We applied latent class growth analysis to estimate membership into trajectory groups based on self-reported sleep duration over time. Analysis of gray matter volumes was carried out using FSL Voxel-Based-Morphometry and white matter microstructure using Tract Based Spatial Statistics. We assessed group differences in cognitive and MRI outcomes using nonparametric permutation testing.

**Results:**

Latent class growth analysis identified four trajectory groups, with an average sleep duration of 5.4 ± 0.2 hours (5%, *N* = 29), 6.2 ± 0.3 hours (37%, *N* = 228), 7.0 ± 0.2 hours (45%, *N* = 278), and 7.9 ± 0.3 hours (13%, *N* = 78). No differences in cognition, gray matter, and white matter measures were detected between groups.

**Conclusions:**

Our null findings suggest that current sleep guidelines that recommend at least 7 hours of sleep per night may not be supported in relation to an association between sleep patterns and cognitive function or brain structure.

**Statement of Significance**

Up to a third of adults report between 6 and 7 hours of sleep per night, and thus fail to meet sleep guidelines which recommend at least 7 hours of sleep per night. Although extreme short sleep (e.g. ≤5 hours per night) has repeatedly been associated with poor cognitive health, it is currently unclear if such relationships subsist for more moderate short-sleep durations. We found no differences in cognitive or structural MRI measures between groups that reported, on average, 5.4 hours, 6.2 hours, 7.0 hours, and 7.9 hours sleep per night over 5 timepoints spanning 28 years. If replicated with longitudinal markers of cognitive health, such null results could challenge the suitability of current sleep guidelines on cognitive outcomes.

## Introduction

Both short- and long-sleep durations are consistently associated with increased mortality and unfavorable health outcomes, including elevated risk of stroke, diabetes, heart disease, and psychiatric disorders [1, 2]. As a result, guidelines published by the National Sleep Foundation [3], recommend between 7 and 9 hours of sleep per night and the American Academy of Sleep Medicine and Sleep Research Society [4] recommends a minimum of 7 hours of sleep per night for adults to promote optimal health and reduce the risk of adverse outcomes.

With regard to cognitive health, meta-analyses have associated extremes in sleep duration (most frequently defined as ≤5 hours or ≥9 hours) with reduced scores for overall cognition, executive function, verbal memory, and working memory [5]. In addition, a small number of longitudinal studies have indicated that an adverse change in sleep duration over time is associated with impaired cognitive performance [6–8].

In order to understand the mechanisms that underlie associations between sleep and cognition, a number of studies have used magnetic resonance imaging (MRI) techniques to examine the relationship between sleep duration and brain structure. Although both short- and long-sleep durations have been linked with increased rates of brain atrophy [9, 10] and markers of white matter structure [11, 12], results have been shown to vary according to the definition of short-, normal-, and long-sleep duration applied [10], and sleep duration has typically only been examined at a single time point. Assessing sleep over an extended period of time has the potential to characterize how sleep habits change with age, and to identify which trajectories are associated with adverse health outcomes.

Here, we examine the relationship between patterns of sleep duration over 28 years and measures of cognition, gray matter volume and white matter microstructure in members of the Whitehall II Imaging Sub-Study. We hypothesize that consistently meeting sleep guidelines (i.e. sleeping at least 7 hours per night) will be associated with higher cognitive scores, greater gray matter volumes, increased fractional anisotropy (FA) and reduced radial diffusivity (RD) values compared with consistently missing the guidelines, or transitioning in and out of the guidelines over time.

## Methods

### Participants

All participants were members of the prospective occupational cohort study, Whitehall II [13] and the Whitehall II Imaging Sub-Study [14]. Ethical approval was obtained from the University of Oxford Central University Research Ethics Committee, and the UCL Medical School Committee on the Ethics of Human Research. Informed written consent was obtained from all participants.

We excluded from analyses participants who were selected based upon depressive symptoms in 2010, reported a history of dementia or neurological illness, displayed significant abnormalities on structural MRI scans, reported current sleep apnea or prescribed medication for a sleep disorder at the time of the MRI scan, had any missing data for cognitive or MRI outcomes, or had missing data at more than one time point for sleep duration. Thus, the analytic sample included 613 participants (Supplementary Material Figure S1).

### Assessment of sleep duration

Sleep duration was assessed with the question “How many hours of sleep do you have on an average week night?” at five previous phases of the Whitehall II Study (1985–88, 1997–99, 2002–04, 2007–09, 2012–13). Participants selected their answers from the following options: “5 hours or less,” “6 hours,” “7 hours,” “8 hours,” or “9 hours or more.” For the purposes of this study, “5 hours or less” was coded as 5 hours and “9 hours or more” was coded as 9 hours.

### Cognitive assessments

Cognition was assessed using a previously described battery of cognitive tests, at the same timepoint as the MRI scan (2012–2016) [14]. With the exception of TMT at Phase 11, these tests had not previously been administered as part of the Whitehall II Study. In line with our previous work [15], cognitive tests were divided into general cognition (Montreal Cognitive Assessment [MoCA] [16]), executive function (digit span: forward, backward, and sequence [17], fluency: letter and category, and trail-making test [TMT]: B [18]), memory (Hopkins Verbal Learning Test Revised [HVLT-R]: total recall, delayed recall, and recognition [19], and Rey-Osterrieth complex figure [RCF]: immediate recall, delayed recall, and recognition [20]) and processing speed (TMT: A [18], digit coding [17], and Cambridge Neuropsychological Test Automated Battery Reaction Time touchscreen task [CANTAB RTI; CANTABeclipse 5.0; Cambridge Cognition Ltd] simple reaction time, choice reaction time, simple movement time, choice movement time [21]). For TMT and CANTAB, signs (positive/negative) were reversed to ensure that higher scores represented a better performance for all tests.

### MRI acquisition and analysis

MRI data were acquired at the Oxford Centre for Functional MRI of the Brain (FMRIB) using a 3-Tesla Siemens Magnetom Verio (Erlangen, Germany) scanner (April 2012–December 2015) with a 32-channel head coil or a 3-Tesla Siemens Magnetom Prisma (June 2015–December 2016), with a 64-channel head-neck coil. Data processing and analysis were carried out using tools from the FMRIB Software Library (http://www.fmrib.ox.ac.uk/fsl) [22, 23]. Gray matter was examined on a voxel-wise basis using FSL-VBM [24] and Tract Based Spatial Statistics (TBSS) was used to examine FA, axial diffusivity (AD), and RD on a voxelwise basis [25]. Full details are provided in Supplementary Material: Text S1.

### Statistical analysis

#### Identifying trajectories of sleep

To identify how sleep patterns changed over time, a set of unconditional latent growth curve models were performed. Several patterns of change were assessed, including: (1) no change, (2) linear change, and (3) quadratic change over time. Variances of the observed sleep variables were constrained to be equal over time. Adequate model fit [26] was determined by a low Akaike information criterion (AIC), Bayesian information criterion (BIC), and sample-size adjusted BIC (SSA-BIC), Tucker-Lewis Index (TLI) ≥ 0.95, Comparative fit indices (CFI) ≥ 0.95 [27], and Root Mean Square Error of Approximation (RMSEA) ≥ 0.10 [28]. As latent growth curve models may not necessarily be sensitive to those with extreme sleep durations, we largely used these models to determine the average pattern of change in sleep patterns over time, which was used to inform the next stage of analysis. As the latent growth curve analysis suggested that a linear or quadratic pattern of change described the observed sleep patterns (Supplementary Material Table S1) better than an intercept-only model, linear and quadratic latent class growth models were examined to evaluate whether individuals could be reliably sub-divided into groups (i.e. “classes”) based on their self-reported hours of sleep. For the latent class growth analyses, the best fit was determined by the model with the lowest AIC, BIC, and SSA-BIC values, entropy ≥ 0.8 and a *p*-value < 0.05 for the Lo-Mendel-Rubin likelihood ratio test (LMR-LRT) and Bootstrap likelihood ratio test (BLRT) [29, 30]. An additional criterion for model selection was that each class was a minimum of 1% of the total sample [29]. We also examined whether between-class differences in the pattern of change improved overall model fit.

Years since the baseline assessment was used as the time scale for both the latent growth curve and latent class growth analyses and these values were divided by either 10 (former [31]) or 100 (latter [32]) to improve model convergence. Restricted Maximum Likelihood with robust standard errors was also used for all models, due to the robustness of this estimator to deviations from normality [33]. The variances of all of the sleep duration items were restricted to be equal across each of the study phases. All LGCM and LCGM analyses were conducted in MPLUS 8 (version 1.6).

#### Assessing associations between sleep trajectories and the aging brain

We employed permutation-based methods for nonparametric testing to examine differences between sleep trajectory groups [34]. Differences between sleep trajectory groups were first examined using *F* tests, and *t*-tests performed when an *F* test was significant (*p* < 0.05).

The FSL tool Permutation Analysis of Linear Models (PALM) was used for the statistical analysis of demographic, cognitive, and global MRI measures. To reduce multiple comparisons for cognitive outcomes, Fisher nonparametric combination (NPC) testing was used to assess the overall *p* values for each cognitive domain [35]. The FSL tool Randomize was used for the voxelwise analysis of MRI data. Five thousand permutations were used, with threshold-free cluster enhancement and family-wise error rate correction applied. Age, sex, and education were included as covariates in the analysis of cognitive data. Age, sex, education, and MRI scanner were included as covariates in the analysis of MRI data.

## Results

### Participants

A total of 613 participants were included in analyses (Supplementary Material Figure S1). Participants included in analyses were not significantly different from participants excluded because of missing data in their age, sex, or education level (Supplementary Material Table S2).

### Sleep trajectories

**Figure 1.**
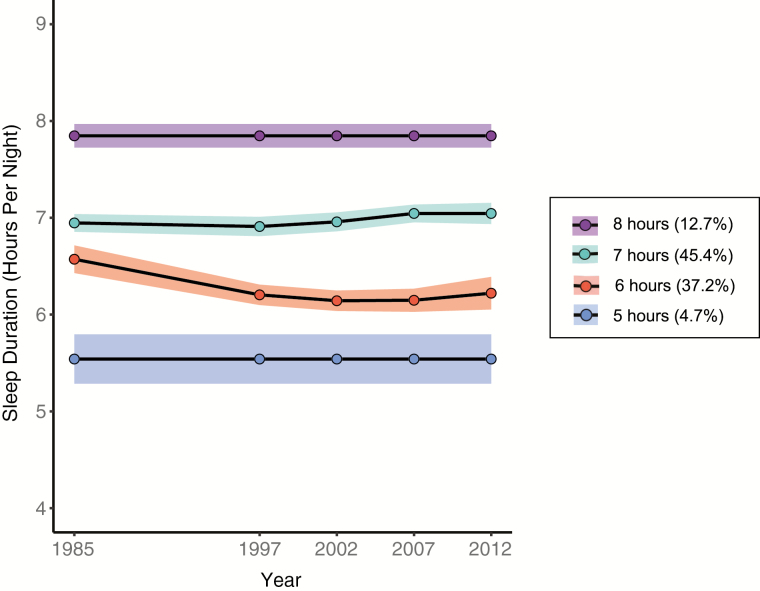
Trajectories identified by latent class growth analyses based on self-reported sleep duration. This data-driven approach suggested that there may be up to four different sleep trajectories identified within the Whitehall II sample, ranging from two subgroups that did not seem to change their hours of sleep over time (groups with average of “8 hours” and “5 hours” of sleep per night) to those that slightly increased (group with average of “7 hours” of sleep) or slightly shortened (“5 hours” group) their sleep over the 28 year follow-up period. Shaded areas represent 95% confidence intervals.

Latent class growth analyses suggested that the sample could be divided into four groups (2 with no change; 2 with quadratic change) based on their self-reported sleep duration, measured over five time points (Figure 1, Supplementary Material, Table S3). Twenty-nine participants (4.73%) were classified into a “5 hours” group, with an average sleep duration across all timepoints of 5.44 ± 0.24 hours. A total of 228 participants (37.19%) were classified into a quadratic “6 hours” group, with duration decreasing from the baseline (1985–1988), and average sleep duration across all timepoints of 6.24 ± 0.26 hours. A total of 278 participants (45.35%) were classified into a quadratic “7 hours” group, with an average sleep duration across all timepoints of 7.02 ± 0.24 hours. A total of 78 participants (12.72%) were classified into an “8 hours” group, with an average sleep duration across all timepoints of 7.87 ± 0.26 hours.

Demographics for each group are presented in Table 1. Groups did not differ in terms of age or sex. Education varied between groups, with the 5-hour group displaying significantly lower levels of education compared with the 6, 7-, and 8-hour groups.

**Table 1.** Demographics and MRI outcomes

	5 hours	6 hours	7 hours	8 hours	*F* test *p*-value
*N* (%)	29 (4.7%)	228 (37.2%)	278 (45.3%)	78 (12.7%)	
Demographics					
Age	69.20 ± 5.38	69.36 ± 4.93	69.62 ± 4.98	70.44 ± 5.40	0.404
Gender—*N* (%) female	10 (34.48)	45 (19.74)	50 (17.99)	12 (15.38)	0.142
Education	2.79 ± 0.86	3.46 ± 1.11	3.59 ± 1.00	3.73 ± 1.09	<0.001*
Tissue types					
GM (%)	38.59 ± 2.16	38.42 ± 1.87	38.43 ± 1.91	38.17 ± 1.99	0.666
WM (%)	38.21 ± 2.52	37.75 ± 2.35	37.87 ± 2.30	37.94 ± 2.30	0.686
CSF (%)	23.21 ± 3.45	23.83 ± 3.11	23.70 ± 3.00	23.89 ± 3.26	0.865
White matter					
FA	0.478 ± 0.018	0.479 ± 0.019	0.479 ± 0.018	0.481 ± 0.021	0.625
AD (×10^3^)	1.084 ± 0.021	1.080 ± 0.023	1.083 ± 0.026	1.082 ± 0.025	0.512
RD (×10^3^)	0.488 ± 0.026	0.485 ± 0.027	0.487 ± 0.028	0.485 ± 0.031	0.625

Figures are means, unless otherwise stated. * 5 < 6, 7, 8 in post hoc *t*-tests. RD: radial diffusivity. Age, gender, education, and scanner were included as covariates in analyses of MRI outcomes. Education was scored on a five-point scale: (1) no qualifications, (2) O-levels or equivalent, (3) A-levels, college certificate or professional qualification, (4) degree, and (5) higher degree. AD = axial diffusivity, , CSF: cerebrospinal fluid, FA = fractional anisotropy, GM = gray matter, WM = white matter.

### Cognitive and MRI outcomes

Descriptive values for all cognitive tests are provided in Supplementary Material Table S4. After adjusting for age, sex, and education, no significant group differences were detected for MoCA scores (*p* = 0.158), executive function (Fisher NPC testing *p* = 0.266), memory (*p* = 0.983), or processing speed (*p* = 0.571).

MRI outcomes are presented in Table 1. In an analysis adjusted for age, sex, education, and scanner, no significant group differences were detected for global measures of percentage GM, percentage WM, percentage CSF, FA, AD, or RD. Voxelwise analysis was also not significant for FSL-VBM (*F* test minimum *p* = 0.358) and TBSS (FA *p* = 0.766, AD *p* = 0.552, RD *p* = 0.700).

## Discussion

The aim of this study was to examine sleep duration trajectories over a 28-year period and their relationship with measures of cognition, gray matter volume, and white matter microstructure. We hypothesized that consistently meeting recommendations for sleep duration (i.e. self-reporting a minimum of 7 hours sleep per night) would be favorably associated with cognition, gray matter volume and white matter microstructure, compared with consistently not meeting the guidelines or transitioning in and out of the guidelines over time. In contrast to our hypotheses, our results did not show any differences in cognitive measures, gray matter volume, or white matter microstructure between different sleep trajectory groups.

To the best of our knowledge, only one study has previously applied latent class growth modeling to examine trajectories of self-reported sleep duration over time within an adult population. In a study of 8,673 Canadian adults, Gilmour et al. identified four sleep trajectory groups with intercepts of 5.57 hours (11% of participants), 6.68 hours (49%), 7.65 hours (37%), and 8.34 hours (2%) [36]. We also identified four trajectory groups, with intercepts of 5.54 hours (5% of participants), 6.57 hours (37%), 6.95 hours (45%), and 7.85 (13%) hours. With regard to shape, the trajectories identified in both studies displayed limited meaningful change over time. For example, average sleep duration differed by less than an hour between timepoints in all groups—indicating that extreme increases or decreases in sleep duration over time are limited in prevalence. Given that our studies differ both in terms of demographics and methods (e.g. sleep duration was assessed over an 8-year time period in Gilmour et al. [36], compared with over 28-years in our study), it is encouraging that our results are broadly complimentary.

Of particular note within our study is that 37% of participants were included in a group with an average sleep duration of 6.2 hours. Guidelines published by the American Society for Sleep Medicine and the Sleep Research Society state that “adults should sleep 7 or more hours per night on a regular basis to promote optimal health” [4]. In addition, the National Sleep Foundation’s guidelines posit that 7–9 hours sleep per night is “recommended” for health and well-being, with less than 6 hours sleep “not recommended” [3]. In these guidelines, 6 hours sleep per night falls in a somewhat gray area between these two groups, and is classified as “may be appropriate.” Relevant to the revision of such guidelines, our study found no evidence to suggest that consistently reporting approximately 6 hours sleep per night is associated with adverse cognitive and MRI outcomes.

Such null findings, however, do not necessarily indicate that sleep duration is not important to cognitive health. Rather, our null findings may reflect the limited number of participants reporting *extremes* in sleep duration within our sample. At each phase, between 92% and 96% of participants reported 6, 7, or 8 hours sleep per night (Supplementary Material Table S5). The group with the shortest sleep duration in our study contained just 5% of participants and had a mean sleep duration of 5.4 hours. The group with the longest sleep duration, which contained 13% of participants, had a mean sleep duration of 7.87 hours—a value that falls within guidelines for recommended sleep durations. Change in sleep duration was also limited within our sample—between 89% and 94% of participants reported change in sleep duration of 0–1 hour between the baseline and subsequent data waves (Supplementary Material Tables S6–S7), and sleep trajectories remained relatively stable over time overall. Significant group differences for cognitive and MRI measures may only become apparent with larger samples of more extreme sleep durations, groups that have often been the focus of cognitive studies to date. For example, in a meta-analysis that reported significant associations between sleep duration and cognitive outcomes, the most common category for short-sleep duration was 5 hours or less (ranging from <4 to ≤6.5 hours), and the most common category for long-sleep duration was 9 hours or more (ranging from ≥8 to ≥11 hours) [5]. Furthermore, although few studies have examined the change in sleep duration over time, Devore et al. reported that women whose sleep duration changed by 2 hours or more in any direction, had worse cognitive outcomes compared with women with no change in sleep duration [8]. In addition, previous studies based on the entire Whitehall II cohort found that adverse changes in sleep duration are associated with poorer cognitive function [6]. There are many reasons that our findings may diverge, despite overlapping samples. These include differences in sample size (5431 vs 631; thereby impacting on power to assess extremes in sleep duration and change), characteristics (see Supplementary Material Table S8 for comparison), number of assessments of sleep duration (2 vs 5 times), and cognitive test battery administered. Therefore, our study does not contradict the hypothesis that extreme short sleep, extreme long sleep, or extreme changes in sleep duration are associated with adverse outcomes; but instead indicates that such groups may not be well represented in small population-based samples.

An alternative explanation for our null results is that it is not sleep duration alone that is associated with cognitive health in aging, but rather a combination of sleep quality and quantity. Indeed, in an overlapping sample, we have previously published that poor sleep *quality* is associated with reduced FA and increased RD within fronto-subcortical regions [15]. In an exploratory post hoc analysis, we divided the 6-hour and 7-hour groups into poor and good sleep quality groups dependent upon their PSQI score at the time of the MRI scan (due to limited sample size, we did not include 5-hour and 8-hour groups in this analysis) (Supplementary Material: Text S2, Table S9). *F* tests showed significant group differences for global FA and voxel-wise RD. The 6-hour good sleep quality group displayed higher global FA and reduced RD in widespread tracts, compared with both the 6-hour poor sleep quality group and 7-hour poor sleep quality group (Figure S2). The 6-hour good sleep quality group also displayed reduced RD compared with the 7-hour good sleep quality group in the corpus callosum (Figure S2). These results indicate that the combination of sleep quality and quantity may be more sensitive to measures of cognitive health in aging. However, it is critical to stress that our measures of sleep quality and quantity are not directly comparable (e.g. quality was measured using a 17-item questionnaire at a single timepoint, duration was measured using a single-item questionnaire at five timepoints). Therefore, these post hoc results should be considered exploratory and require independent replication.

Our study has a number of strengths, including the availability of sleep duration data at five points spanning 28 years prior to cognitive and MRI assessment, which allowed us to examine sleep trajectories over time. A major limitation of our analysis was the reliance on a single-item self-report of sleep duration, in which participants could only report their sleep durations in discrete categories (i.e. “5 hours or less,” “6 hours,” “7 hours,” “8 hours,” or “9 hours or more”). Sleep duration may be more sensitively measured if sleep was measured in hours and minutes and if there were no lower or upper thresholds for sleep duration. A further limitation is that participants were asked to report their sleep duration only on an “average week night.” The discrepancy between weeknight and weekend sleep duration is common in working-age populations and there is debate regarding whether long weekend sleep can compensate for short weeknight sleep for health outcomes such as mortality [37], weight, and insulin sensitivity [38]. In the Whitehall II study, the agreement between self-reported and accelerometer-measured total sleep duration was slightly higher in weekdays (kappa = 0.37, 95% CI 0.34–0.40) than weekend days (kappa = 0.33, 95% CI 0.31–0.36) [39]. Further research is needed to examine the long-term effects of weekend recovery sleep on cognition. Sleep duration is also often overestimated in self-reported compared to objective studies [40–42]. For example, within the Sleep Heart Health Study of 2,113 adults at a mean age of 67 years, morning self-estimated sleep time and total sleep time measured by polysomnography were estimated as 379 and 363 minutes, respectively, with a weak correlation of *r* = 0.16 between the measures [41]. Self-reported total sleep duration and sleep duration assessed using a wrist-worn accelerometer were moderately related in the Whitehall II study of 4,094 adults aged 60–83 (kappa 0.39, 95% CI 0.36–0.42) [39]. Importantly, differences between measurements may impact upon observed relationships with cognitive outcomes. For example, the Sleep Study of the National Social Life, Health, and Aging Project (NSHAP), a nationally representative cohort of older US adults (2010–2015), found that actigraphic measures of sleep disruption were associated with worse cognition and higher odds of 5-year cognitive decline but there was no association for self-reported sleep [43]. As self-reported measures of sleep duration correlate well with daily sleep diaries [44], are the mainstay of population-based cohort studies, and are the focus of sleep guidelines, further studies using both objective and self-report measures of sleep duration to examine cognitive and MRI outcomes are needed. Furthermore, our findings have limited generalizability, given that the Whitehall participants have relatively high educational attainment which might contribute to increased cognitive reserve. As a result, we may have underestimated the long-term neurocognitive effects associated with unfavorable sleep patterns in subpopulations of low educational attainment, who may be more sensitive to the detrimental health effects of sleep disturbance. We were also unable to rule out potential selection bias; it is plausible that those with the greatest cognitive decline and/or most extreme sleep durations were less likely to return for a follow-up assessment.

## Conclusion

Due to the results from previous observational studies, we hypothesized that consistently meeting sleep guidelines (i.e. sleeping at least 7 hours per night) would be associated with improved cognition, increased gray matter volumes and improved white matter microstructure (increased FA and reduced RD) compared with consistently missing the guidelines, or transitioning in and out of the guidelines over time. However, such hypotheses were not supported by our results, as we found that sleep duration remained relatively stable over 28 years and that there was no evidence of differences in cognition or MRI findings between sleep trajectory groups. If replicated, such null results could challenge the suitability of current sleep guidelines on cognitive outcomes and open doors to new directions in research regarding the appropriateness of considering sleep duration and quality together.

## Funding

This research was supported by the UK Medical Research Council (MRC, G1001354) and the NIHR Oxford Health Biomedical Research Centre. The Wellcome Centre for Integrative Neuroimaging is supported by core funding from the Wellcome Trust (203139/Z/16/Z). Whitehall II was supported by the MRC (K013351, R024227); the British Heart Foundation (PG/11/63/29011 and RG/13/2/30098); the National Heart, Lung, and Blood Institute (R01HL036310); the National Institute on Aging, National Institute of Health (NIA, R01AG056477, R01AG034454); and the Economic and Social Research Council (ES/J023299/1). JZ received funding from the Global Brain Health Institute (GBHI), Alzheimer’s Association, and Alzheimer’s Society (GBHI ALZ UK-19-585532). MK is supported by the MRC (R024227, S011676), NIA (R01AG056477), and the Academy of Finland (311492). NF was funded by the NIHR Oxford Health Biomedical Research Centre. YL is supported by the National Institute on Aging (NIA) (1K99AG056598), and received funding from GBHI, Alzheimer’s Association, and Alzheimer’s Society (GBHI ALZ UK-19-591141).

## Supplementary Material

zsz290_suppl_Supplementary_Material
