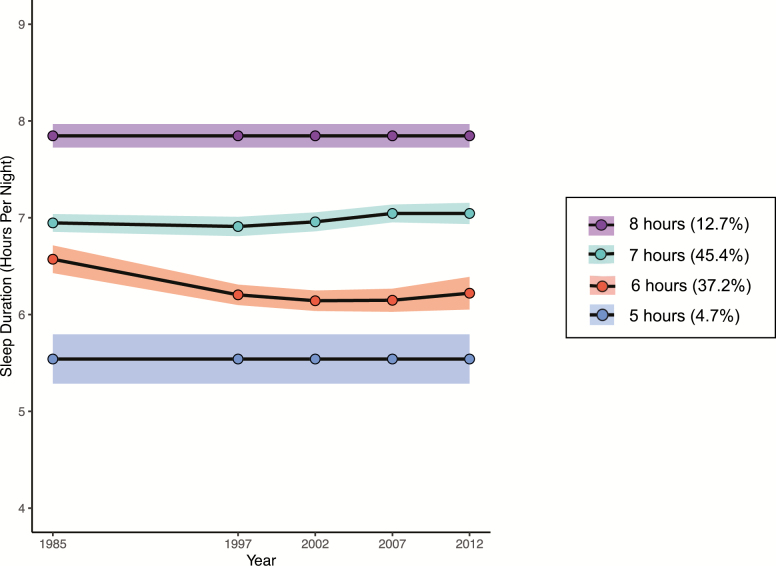# Corrigendum to: Sleep duration over 28 years, cognition, gray matter volume, and white matter microstructure: a prospective cohort study

**DOI:** 10.1093/sleep/zsaa028

**Published:** 2020-03-19

**Authors:** Jennifer Zitser, Melis Anatürk, Enikő Zsoldos, Abda Mahmood, Nicola Filippini, Sana Suri, Yue Leng, Kristine Yaffe, Archana Singh-Manoux, Mika Kivimaki, Klaus Ebmeier, Claire Sexton

In the article “Sleep duration over 28 years, cognition, gray matter volume, and white matter microstructure: a prospective cohort study” (*SLEEP*, doi:10.1093/sleep/zsz290), Figure 1 was incorrect. This has been corrected in the original article.