# Supplementary material for: Sleep duration over 28 years, cognition, gray matter volume, and white matter microstructure: a prospective cohort study
Source: Sleep. 2020 Jan 6;43(5):zsz290. doi: 10.1093/sleep/zsz290 (PMC7215267; doi:10.1093/sleep/zsz290)
Supplement: zsz290_suppl_Supplementary_Material [file zsz290_suppl_supplementary_material.docx]

**Supplementary Material for:**

**Sleep Duration over 28 Years, Cognition, Grey Matter Volume and White Matter Microstructure:**

**A Prospective Cohort Study**

Jennifer Zitser,^1,2^ Melis Anatürk,^3^ Enikő Zsoldos,^3,4,5^ Abda Mahmood,^3^ Nicola Filippini,^4^ Sana Suri,^4^ Yue Leng,^1,6^ Kristine Yaffe,^7^ Archana Singh-Manoux,^8,9^ Mika Kivimaki,^9^ Klaus Ebmeier,^3^ Claire Sexton^1,4^

^1^Global Brain Health Institute, Memory and Aging Center, Department of Neurology, University of California San Francisco, CA, USA

^2^Movement Disorders Unit, Department of Neurology, Tel Aviv Sourazky Medical Center, affiliated to the Sackler Faculty of Medicine, Tel-Aviv University, Israel

^3^Department of Psychiatry, University of Oxford, Oxford, UK

^4^Oxford Centre for Human Brain Activity, Wellcome Centre for Integrative Neuroimaging, Department of Psychiatry, University of Oxford

^5^FMRIB, Wellcome Centre for Integrative Neuroimaging, University of Oxford

^6^Department of Psychiatry, University of California San Francisco, CA, USA

^7^Department of Psychiatry, Neurology and Epidemiology, University of California San Francisco, CA, USA

^8^Université de Paris, Inserm U1153, Epidemiology of Ageing and Neurodegenerative diseases, Paris, France

^9^Department of Epidemiology and Public Health, University College London, London, UK

Corresponding author:

Claire Sexton, [claire.sexton@gbhi.org](mailto:claire.sexton@gbhi.org)

Department of Psychiatry, University of Oxford, Warneford Hospital, Oxford, OX3 7JX

**Supplementary Material**

**Table S1. Results for latent growth curve analyses.**

|  | **AIC** | **BIC** | **SSA-BIC** | **CFI** | **TLI** | **RMSEA (CI)** |
| --- | --- | --- | --- | --- | --- | --- |
| Intercept only | 6594.104 | 6607.359 | 6597.835 | 0.82 | 0.894 | 0.123 (0.107 - 0.14) |
| **Linear** | **6498.239** | **6524.749** | **6505.7** | **0.924** | **0.946** | **0.088 (0.07 - 0.107)** |
| **Quadratic** | **6443.906** | **6488.089** | **6456.341** | **0.983** | **0.983** | **0.049 (0.025 - 0.074)** |

**Abbreviations:** AIC - Akaike Information Criterion; BIC - Bayesian Information Criterion; CFI - Comparative Fit Index; CI - Confidence Intervals; RMSEA - Root Mean Square Error of Approximation; SSA-BIC - Sample size adjusted Bayesian Information Criterion; TLI - Tucker-Lewis Index.

**Figure S1. Attrition of participants.**

**Text S1. MRI Acquisition and Analysis**

T_1_-weighted images were acquired using a three-dimensional rapid gradient echo sequence (repetition time 2530ms, echo time 7.37 ms (Verio) or 2.19 ms (Prisma), flip angle 7˚, field of view 256 mm, voxel size 1.0 x 1.0 x 1.0 mm). T1-weighted images were processed using fsl_anat (http://fsl.fmrib.ox.ac.uk/fsl/fslwiki/fsl_ anat), grey matter, white matter and cerebrospinal fluid percentages were calculated using the FMRIB Automated Segmentation Tool (FAST) [1], and grey matter was examined on a voxel-wise basis using FSL-VBM [2]. First, segmented grey matter was registered to MNI 152 standard space using non-linear registration [3]. These images were then averaged and flipped along the x-axis to create a left-right ﻿symmetric, study-specific grey matter template. Next, all native grey matter images were non-linearly registered to the study specific template and modulated to correct for local expansion or contraction due to the non-linear component of the spatial transformation. The modulated grey matter images were then smoothed with an isotropic Gaussian kernel with sigma of 3 mm.

Diffusion-weighted images were collected using echo-planar imaging (repetition time 8900 ms, echo time 91.2 ms (Verio) or 91 ms (Prisma), field of view 192 mm, voxel size 2.0 x 2.0 x 2.0 mm), with 60 diffusion weighted directions (b-value 1500s/mm^2^), five non-diffusion weighted images (b-value 0s/mm^2^) and one b0 volume collected in the reversed phase encoded direction.﻿ ﻿Corrections for head motion, susceptibility and eddy- current induced distortions were performed using the FSL tools topup [4] and eddy [5,6]. Slices were classified as outliers and replaced if the signal was more than three standard deviations from the Gaussian process predicted slice. If over 10 slices were identified as outliers within a volume, the volume was removed. If more than five volumes were removed, than the scan was excluded from analyses. DTIFit, part of FMRIB’s Diffusion Toolbox, was used to fit a diffusion tensor model to the raw diffusion data, before Tract Based Spatial Statistics (TBSS) was used to examine fractional anisotropy (FA), axial diffusivity (AD) and radial diffusivity (RD) on a voxelwise basis [7]. All participants' FA, AD, and RD data were aligned into a common space using FMRIB's Nonlinear Registration Tool (FNIRT) [3,8]. Next, the mean FA image was created and thinned to create a mean FA skeleton representing the centres of all tracts common to the group. The threshold for the mean FA skeleton was set at 0.2, resulting in a mask of 119,883 voxels. Each participant’s aligned FA, AD and RD data was then projected onto the tract skeleton. Global measures of mean FA, AD and RD were calculated across the white matter skeleton.

**Table S2. Comparison of participants included and those excluded due to missing data.** Values are mean ± standard deviation unless stated otherwise.

^a^ corrected for multiple contrasts

|  | **Complete Data**  **(n=613)** | **Missing Data**  **(n=78)** | **Cohen’s d** | **p^a^** |
| --- | --- | --- | --- | --- |
|  |  |  |  |  |
| Age (years) | 69.61 ± 5.03 | 70.35 ± 5.14 | 0.15 | 0.205 |
| Sex (N females, %) | 117 (19.09%) | 20 (25.64%) | 0.16 | 0.173 |
| Education | 3.52 ± 1.06 | 3.38 ± 1.19 | -0.13 | 0.269 |

﻿Education was scored on a five-point scale: (1) no qualifications, (2) O-levels or equivalent, (3) A-levels, college certificate or professional qualification, (4) degree, (5) higher degree.

**Table S3. Results for latent class growth analyses.**

|  |  |  |  |  | **N of Participants Per Class Based on Most Likely Class Membership** | | | | | |  |  |
| --- | --- | --- | --- | --- | --- | --- | --- | --- | --- | --- | --- | --- |
|  | **AIC** | **BIC** | **SSA- BIC** | **Entropy** | **Class 1** | **Class 2** | **Class 3** | **Class 4** | **Class 5** | **Class 6** | **LMR - LRT**  **p-value** | **BLRT**  **p-value** |
| **Linear latent class growth analyses** | | |  |  |  |  |  |  |  |  |  |  |
| *2 classes* | 6817.364 | 6843.874 | 6824.825 | 0.769 | 328 | 285 |  |  |  |  | 0 | 0 |
| *3 classes* | 6604.122 | 6643.888 | 6615.315 | 0.782 | 205 | 326 | 82 |  |  |  | 0 | 0 |
| *4 classes* | 6548.592 | 6601.613 | 6563.515 | 0.783 | 223 | 28 | 78 | 284 |  |  | 0 | 0 |
| *5 classes* | 6528.152 | 6594.428 | 6546.806 | 0.786 | 217 | 78 | 16 | 273 | 29 |  | 0.0021 | 0 |
| *6 classes* | 6510.948 | 6590.479 | 6533.332 | 0.785 | 76 | 27 | 201 | 275 | 23 | 11 | 0.0122 | 0 |
| **Quadratic latent class growth analyses** | | |  |  |  |  |  |  |  |  |  |  |
| *2 classes* | 6789.963 | 6825.31 | 6799.912 | 0.773 | 286 | 327 |  |  |  |  | 0 | 0 |
| *3 classes* | 6573.914 | 6626.935 | 6588.837 | 0.785 | 326 | 82 | 205 |  |  |  | 0 | 0 |
| *3 classes^a^* | 6580.509 | 6624.693 | 6592.945 | 0.794 | 211 | 78 | 324 |  |  |  | 0 | 0 |
| *4 classes* | 6516.936 | 6587.63 | 6536.833 | 0.786 | 78 | 28 | 278 | 229 |  |  | 0.0009 | 0 |
| ***4 classes^b^*** | **6530.058** | **6583.078** | **6544.981** | **0.788** | **78** | **278** | **29** | **228** |  |  | **0.0002** | **0** |
| *5 classes* | 6489.001 | 6577.368 | 6513.873 | 0.784 | 32 | 78 | 29 | 202 | 272 |  | 0.0634 | 0 |
| 6 classes | 6470.153 | 6576.194 | 6499.998 | 0.789 | 35 | 193 | 27 | 12 | 80 | 266 | 0.0083 | 0 |

**Abbreviations:** AIC = Akaike Information Criterion; BIC = Bayesian Information Criterion; BLRT = Bootstrap likelihood ratio test; LMR-LRT = Lo-Mendel-Rubin likelihood ratio test; SSA-BIC = Sample size adjusted Bayesian Information Criterion.

^a^ As the means for the linear and quadratic slope of class 2 were non-significant, these parameters were constrained to 0.

^b^ As the means for linear and quadratic slope were non-significant for classes 1 and 4, these parameters were restricted to 0 for the two classes.

**Table S4. Results for the cognitive assessment.** Values are mean ± standard deviation unless stated otherwise.

|  | **5 hours** | **6 hours** | **7 hours** | **8 hours** |
| --- | --- | --- | --- | --- |
|  |  |  |  |  |
| **General Cognition** |  |  |  |  |
| MoCA | 26.66 ± 2.29 | 27.20 ± 2.14 | 27.46 ± 2.14 | 27.09 ± 2.48 |
|  |  |  |  |  |
| **Executive Function** |  |  |  |  |
| Fluency: Category | 21.97 ± 3.55 | 22.73 ± 5.63 | 22.53 ± 5.16 | 22.32 ± 6.47 |
| Fluency: Letter | 14.14 ± 4.47 | 15.66 ± 4.51 | 15.83 ± 4.19 | 16.55 ± 5.07 |
| Trail Making Test: B | -59.55 ± 24.44 | -67.95 ± 38.66 | -64.91 ± 28.55 | -63.24 ± 26.71 |
| Digit Span: Forward | 10.52 ± 2.29 | 10.99 ± 2.22 | 11.16 ± 2.23 | 11.41 ± 2.52 |
| Digit Span: Backward | 9.69 ± 2.38 | 9.63 ± 2.46 | 9.58 ± 2.44 | 10.19 ± 2.51 |
| Digit Span: Sequence | 9.28 ± 2.71 | 10.00 ± 2.47 | 10.24 ± 2.48 | 9.82 ± 2.07 |
|  |  |  |  |  |
| **Memory** |  |  |  |  |
| HVLT-R: Total Recall | 28.10 ± 4.80 | 27.68 ± 4.58 | 27.96 ± 4.49 | 27.55 ± 3.99 |
| HVLT-R: Delayed Recall | 9.21 ± 3.30 | 9.46 ± 2.47 | 9.34 ± 2.50 | 9.37 ± 2.47 |
| HVLT-R: Recognition | 10.83 ± 1.56 | 10.81 ± 1.48 | 10.83 ± 1.35 | 10.79 ± 1.27 |
| RCF: Immediate | 16.22 ± 8.03 | 15.96 ± 6.57 | 15.84 ± 6.08 | 16.23 ± 6.98 |
| RCF: Delayed | 15.31 ± 7.63 | 15.82 ± 6.31 | 15.46 ± 5.76 | 15.72 ± 6.34 |
| RCF: Recognition | 8.55 ± 1.90 | 8.65 ± 1.79 | 8.46 ± 2.02 | 8.47 ± 1.84 |
|  |  |  |  |  |
| **Processing Speed** |  |  |  |  |
| Trail Making Test: A | -32.00 ± 10.76 | -30.82 ± 10.55 | -30.17 ± 10.85 | -28.63 ± 10.10 |
| Digit Coding | 62.41 ± 11.71 | 64.04 ± 13.07 | 62.49 ± 12.78 | 64.51 ± 14.75 |
| CANTAB: Simple Reaction Time | -323.72 ± 108.51 | -292.41 ± 56.82 | -296.82 ± 67.17 | -298.91 ± 61.96 |
| CANTAB: Choice Reaction Time | -334.95 ± 69.44 | -330.43 ± 43.44 | -330.37 ± 47.65 | -337.94 ± 50.15 |
| CANTAB: Simple Movement Time | -276.17 ± 96.34 | -272.20 ± 82.41 | -274.01 ± 83.28 | -269.11 ± 84.83 |
| CANTAB: Choice Movement Time | -281.88 ± 93.37 | -291.89 ± 82.50 | -291.75 ± 70.19 | -287.53 ± 77.39 |

**Abbreviations:** HVLT-R - Hopkins Verbal Learning Test Revised; RCF - Rey Complex Figure

**Table S5. Sleep Duration.**

Number (percentage) of participants reporting each category of sleep duration per phase.

|  | 5 hours or less | 6 hours | 7 hours | 8 hours | 9 hours or more |
| --- | --- | --- | --- | --- | --- |
| Phase 1 | 19 (3.10%) | 164 (26.80%) | 327 (53.43%) | 92 (15.03%) | 10 (1.63%) |
| Phase 5 | 32 (5.49%) | 218 (37.39%) | 250 (42.88%) | 77 (13.21%) | 6 (1.03%) |
| Phase 7 | 43 (7.15%) | 196 (32.61%) | 272 (45.26%) | 82 (13.64%) | 8 (1.33%) |
| Phase 9 | 39 (6.25%) | 196 (32.24%) | 244 (40.13%) | 119 (19.57%) | 11 (1.81%) |
| Phase 11 | 41 (6.70%) | 183 (29.90%) | 251 (41.01%) | 124 (20.26%) | 13 (2.12%) |

**Table S6. Change in Sleep Duration from Baseline**

Number (percentage) of participants for each category of change in sleep duration from baseline.

|  | - 3 hours | -2 hours | -1 hour | 0 hours | 1 hour | 2 hours | 3 hours |
| --- | --- | --- | --- | --- | --- | --- | --- |
| Phase 1 - 5 | 3 (0.52%) | 23 (3.95%) | 158 (27.15%) | 304 (52.23%) | 86 (14.78%) | 4 (0.69%) | 4 (0.69%) |
| Phase 1 - 7 | 3 (0.50%) | 28 (4.67%) | 172 (28.67%) | 276 (46.00%) | 105 (17.50%) | 16 (2.67%) | 0 (0.00%) |
| Phase 1 - 9 | 1 (0.16%) | 30 (4.94%) | 152 (25.04%) | 274 (45.14%) | 124 (20.43%) | 25 (4.12%) | 1 (0.16%) |
| Phase 1 - 11 | 5 (0.82%) | 30 (4.91%) | 137 (22.42%) | 280 (45.83%) | 131 (21.44%) | 26 (4.26%) | 2 (0.33%) |

**Table S7. Participants who displayed no change in sleep duration over time**

Number (percentage) of participants for each category of change in sleep duration from baseline.

|  | Sleep Trajectory Group | | | |
| --- | --- | --- | --- | --- |
|  | 5 hours | 6 hours | 7 hours | 8 hours |
| Reported “5 hours or less” on all available timepoints | 4 (14%) | 0 | 0 | 0 |
| Reported “6 hours” on all available timepoints | 0 | 50 (22%) | 0 | 0 |
| Reported “7 hours” on all available timepoints | 0 | 0 | 88 (32%) | 0 |
| Reported “8 hours” on all available timepoints | 0 | 0 | 0 | 18 (23%) |
| Reported “9 hours or more” on all available timepoints | 0 | 0 | 0 | 0 |

**Table S8. Comparison of sample in Ferrie et al 2011** [9] **and the current sample.**

|  | **Ferrie et al 2011**[9] | **Zitser et al** |
| --- | --- | --- |
|  |  |  |
| Sample Size | 5431 | 613 |
| Gender (Female) | 1459 (26.86%) | 117 (19.09%) |
|  |  |  |
| **Sleep Duration at Phase 7: N (%)** |  |  |
| 5 hours or less | 417 (7.68%) | 43 (7.15%) |
| 6 hours | 1740 (32.04%) | 196 (32.61%) |
| 7 hours | 2264 (41.69%) | 272 (45.26%) |
| 8 hours | 897 (16.52%) | 82 (13.64%) |
| 9 hours or more | 113 (2.08%) | 8 (1.33%) |
|  |  |  |
| **Cognition at Phase 7: Range** |  |  |
| Memory | 0 – 18 | 1 – 14 |
| Reasoning | 12 – 65 | 16 – 64 |
| Vocabulary | 1 – 33 | 5 – 32 |
| Phonemic fluency | 3 – 47 | 2 – 27 |
| Semantic fluency | 2 – 34 | 7 – 32 |
| MMSE | 18 – 30 | 24 – 30 |

**Text S2. Sleep Duration and Sleep Quality**

﻿Sleep quality was assessed at the same time-point as the MRI scan using the self-rated questionnaire, the Pittsburgh Sleep Quality Index (PSQI) [10]. A threshold of 6 used to classify participants as good sleepers (< 6) or poor sleepers (≥ 6).[10] Differences in demographics are presented in Table S7. For cognitive outcomes, after covarying for age, gender and education level, there were no significant group differences in MoCA (p = 0.432), executive function (p = 0.721), memory (p = 0.811), or processing speed (p = 0.191). For global MRI outcomes, there were no significant group differences in GM % (p = 0.131), WM % (p = 0.703), CSF % (p = 0.923), AD (p = 0.120) or RD (p = 0.052). There was a significant group difference for FA (p = 0.497), with post-hoc t-tests showing significantly higher FA values in the 6 hour good sleep quality group compared with both the 6 hour poor sleep quality group and the 7 hour poor sleep quality group (Figure S2). In voxel-wise analyses, after covarying for age, gender, education level, and scanner, voxelwise analysis was not significant for FSL-VBM (minimum p = 0.315), or TBSS analysis of FA (p = 0.109) or AD (p = 0.074). An F-test of RD was significant (p = 0.043), with significant regions in subsequent t-tests displayed in Figure S3.

**Table S9. Sleep duration and sleep quality (good or poor quality).** Values are mean ± standard deviation unless specified otherwise.

|  | **6G**  **(n 109)** | **6P**  **(n 119)** | **7G**  **(n 219)** | **7P**  **(n 59)** | **F Test**  **P value** | **T Tests with**  **P < 0.05** |
| --- | --- | --- | --- | --- | --- | --- |
|  |  |  |  |  |  |  |
| **Demographics** |  |  |  |  |  |  |
| Age | 68.32 ± 4.48 | 70.31 ± 5.15 | 69.67 ± 4.92 | 69.44 ± 5.22 | 0.025 | 6G < 6P, 7G |
| Gender - N (%) Female | 23 (21.10) | 22 (18.49) | 29 (13.24) | 21 (35.59) | 0.001 | 7P > 6G, 6P, 7G  6G > 7G |
| Education | 3.39 ± 1.13 | 3.51 ± 1.10 | 3.67 ± 0.98 | 3.31 ± 1.05 | 0.035 | 6G, 7P < 7G |

N/A: Not applicable (i.e. T tests were not performed as the overall F test was not significant). Age, gender, education and scanner were included as covariates in analyses of MRI outcomes. Education was scored on a five-point scale: (1) no qualifications, (2) O-levels or equivalent, (3) A-levels, college certificate or professional qualification, (4) degree, (5) higher degree. 6G and 7G stand for 6- and 7-hour good sleeper. 6P and 7P stand for 6- and 7-hour poor quality sleepers.

**Figure S2. Global differences in DTI metrics.**

* indicates significant post-hoc t-tests (p<0.05)

**Figure S3. Voxelwise group differences in radial diffusivity.**

﻿Localization of group differences in radial diffusivity between 6 hour good sleep quality (6G), 6 hour poor sleep quality (6P), 7 hour good sleep quality (7G) and 7 hour poor sleep quality (7P) groups. Voxels displaying a significant difference in RD are displayed in blue, dilated for illustrative purposes using tbss_fill, and overlaid on a green ﻿skeleton. Age, sex, education and scanner were included as covariates, with significance threshold set at P<0.05, corrected for multiple comparisons across voxels.

**References**

1. Zhang Y, Brady M, Smith S. Segmentation of brain MR images through a hidden Markov random field model and the expectation-maximization algorithm. IEEE Trans Med Imaging [Internet]. 2001 Jan [cited 2019 May 21];20(1):45–57. Available from: http://www.ncbi.nlm.nih.gov/pubmed/11293691

2. Douaud G, Smith S, Jenkinson M, Behrens T, Johansen-Berg H, Vickers J, et al. Anatomically related grey and white matter abnormalities in adolescent-onset schizophrenia. Brain [Internet]. 2007 Sep [cited 2013 May 24];130(Pt 9):2375–86. Available from: http://www.ncbi.nlm.nih.gov/pubmed/17698497

3. Andersson, Jenkinson M, Smith S. Non-linear registration, aka spatial normalisation [Internet]. FMRIB technical report. 2007. p. TR07JA2. Available from: www.fmrib.ox.ac.uk/analysis/techrep

4. Andersson JLR, Skare S, Ashburner J. How to correct susceptibility distortions in spin-echo echo-planar images: application to diffusion tensor imaging. Neuroimage. 2003;20(2):870–88.

5. Andersson JLR, Sotiropoulos SN. An integrated approach to correction for off-resonance effects and subject movement in diffusion MR imaging. Neuroimage [Internet]. 2016 Jan 15 [cited 2015 Dec 21];125:1063–78. Available from: http://www.pubmedcentral.nih.gov/articlerender.fcgi?artid=4692656&tool=pmcentrez&rendertype=abstract

6. Andersson JLR, Graham MS, Zsoldos E, Sotiropoulos SN. Incorporating outlier detection and replacement into a non-parametric framework for movement and distortion correction of diffusion MR images. Neuroimage. 2016;

7. Smith SM, Jenkinson M, Johansen-Berg H, Rueckert D, Nichols TE, Mackay CE, et al. Tract-based spatial statistics: voxelwise analysis of multi-subject diffusion data. Neuroimage [Internet]. 2006 Jul 15 [cited 2012 Nov 3];31(4):1487–505. Available from: http://www.ncbi.nlm.nih.gov/pubmed/16624579

8. Andersson J, Jenkinson M, Smith S. Non-linear optimisation [Internet]. 2007. p. TR07JA1. Available from: http://www.fmrib.ox.ac.uk/analysis/techrep/

9. Ferrie JE, Shipley MJ, Akbaraly TN, Marmot MG, Kivimäki M, Singh-Manoux A. Change in sleep duration and cognitive function: findings from the Whitehall II Study. Sleep [Internet]. 2011 May [cited 2013 Apr 30];34(5):565–73. Available from: http://www.pubmedcentral.nih.gov/articlerender.fcgi?artid=3079935&tool=pmcentrez&rendertype=abstract

10. Buysse DJ, Reynolds CF, Monk TH, Berman SR, Kupfer DJ. The Pittsburgh Sleep Quality Index: a new instrument for psychiatric practice and research. Psychiatry Res [Internet]. 1989 May [cited 2011 Aug 17];28(2):193–213. Available from: http://www.ncbi.nlm.nih.gov/pubmed/2748771
